# Supplementary material for: Maximum Entropy, Word-Frequency, Chinese Characters, and Multiple Meanings
Source: PLoS One. 2015 May 8;10(5):e0125592. doi: 10.1371/journal.pone.0125592 (PMC4425542; doi:10.1371/journal.pone.0125592)
Supplement: S1 Data Set — (ZIP) [file pone.0125592.s001.zip › Data description.pdf]

Data description

The following table lists the text data used in this paper. In order to avoid copyright infringement, the words/characters are randomly placed in the files.

File name	Book name	Author	Language
data-aq-cc	A Q Zheng Zhuan	Xun Lu	Chinese (characters)
data-aq-cw	A Q Zheng Zhuan	Xun Lu	Chinese (words)
data-pf-cc	Ping Fan De Shi Jie	Yao Lu	Chinese (characters)
data-pf-cw	Ping Fan De Shi Jie	Yao Lu	Chinese (words)
data-man-cc	The Man in a Case	A. Chekhov	Chinese (characters)
data-man-ru	The Man in a Case	A. Chekhov	Russian
data-man-en	The Man in a Case	A. Chekhov	English
data-sea-cc	The Old Man and the Sea	E. Hemingway	Chinese (characters)
data-sea-ru	The Old Man and the Sea	E. Hemingway	Russian
data-sea-en	The Old Man and the Sea	E. Hemingway	English
data-hp-cc	Harry Potter	J. K. Rowling	Chinese (characters)
data-hp-en	Harry Potter	J. K. Rowling	English
data-tess-en	Tess of d'Urbervilles	T. Hardy	English
